# Potentially hazardous waste produced at home

**DOI:** 10.1186/1755-7682-6-27

**Published:** 2013-06-27

**Authors:** Loide Corina Chaves, Ligia Mara Daros de Campos, Rosangela Filipini, Luiz Carlos de Abreu, Vitor E Valenti, Ligia Ajaime Azzalis, Virginia Berlanga Campos Junqueira, Dayse F Sena, Flávia C Goulart, Fernando Luiz Affonso Fonseca

**Affiliations:** 1 Department of Morphology and Physiology, School of Medicine of ABC, Av. Príncipe de Gales, n.821, Santo Andre, SP, Brazil; 2 Department of Speech Language and Hearing Therapy, Faculty of Philosophy and Sciences, UNESP, Av. Hygino Muzzi Filho, 737, Marília, SP 17525-900, Brazil; 3 Department of Pharmacy and Biochemistry Federal university of São Paulo, UNIFESP, Rua Artur Riedel, 275, Diadema, SP 09972-270, Brazil; 4 Instituto de Medicina Integral Prof. Fernando Figueira, Rua dos Coelhos, 300, Boa Vista, PE 50070-550, Brasil

**Keywords:** Medical waste, Housing, Environmental health

## Abstract

**Background:**

The purpose of this study was to identify the sources of waste generation household consisting of biological material and to investigate the knowledge presented by those responsible for the generation of waste in the home environment on the potential health risk human and environmental.

**Method:**

It is a quantitative survey performed in Parque Capuava, Santo André (SP). The questionnaire was administered by the community employers and nursing students during the consultation with nursing supervision through interview question/answer. The exclusion criteria were patients who were not in the area served by the Basic Health Unit which covers the area of Pq Capuava. The sample was consisted of 99 persons and the data collection a questionnaire was used.

**Results:**

We observed that 63.3% of people said to use disposables, with the majority (58.7%) of these use the public collection as the final destination of these materials. It was reported that 73.7% of those surveyed reported having knowledge about the risk of disease transmission. Public awareness of the importance of proper packaging and disposal of potentially hazardous household waste may contribute significantly to the preservation of human and environmental health and this procedure can be performed and supervised by professional nurses.

**Conclusion:**

We suggest implementation of workshops for community health workers and the general population in order to enhance their knowledge about the storage and disposal of potentially infectious waste generated at home, thereby reducing the potential risk of disease transmission by improper management.

## Background

The production of solid waste in a community, including the home has increased in recent years due to modern technological development, coupled with the uncontrolled cities growth [1]. This situation has attracted particular attention due to the volume and variety of waste generated, particularly those with high concentrations of potentially infectious microorganisms that make up such waste.

Most of the waste generated at home consists of food scraps like fruit peels, vegetables, spoiled products, newspapers and magazines, bottles, packaging in general, toilet paper, disposable diapers and a wide variety of other items, including products which may be toxic [2] However, the study of Menezes [3], whose aim was to investigate the co-disposal of municipal solid waste and waste of health services (WHS) in landfills in Belo Horizonte, some components have been identified that have both biological risk waste, such as *Clostridium perfringes, enterococci and fecal coliform, Pseudomonas aeruginosa* and *Staphylococcus aureus,* including strains of *Pseudomonas aeruginosa* and *Staphylococcus aureus* multiresistant to antimicrobials.

To potentially infectious waste generated at home, such as syringes used by diabetics, drill-cutting waste generated by drug users, as well as toilet paper, tampons, disposable diapers, wipes and bandages that may be present blood, exudates and secretions [1-4], there is still no differentiated collection, so the final destination is the municipal waste.

Therefore, household waste and potentially hazardous when disposed improperly packaged may present a potential risk to public health, particularly those who are directly involved in handling such as service workers and public collection of people living on waste recycling.

Therefore, the interest in developing this study, it should be a concern to investigate how handled potentially infectious waste generated at home are packaged and the impact on human and environmental health.

Thus, we aimed to identify the sources generating household waste consisting of biological material in Capuava Park in Santo André and investigate the knowledge presented by the individuals responsible for the generation of wastes consisting of biological material in the home environment.

## Method

This is a descriptive and quantitative research, data were obtained through field research and literature review. The survey was conducted in Capuava park located in Santo André, São Paulo State. The population consisted of persons residing in the Park Capuava treated at the Health Centre of the Faculty of Medicine of ABC (FMABC). This institution provides primary care services to the region that corresponds to Capuava core areas Capuava Housing (slum), Park and Garden Capuava Rina.

The sample was composed by 99 selected people. We identified the micro Capuava areas located in the Park, as well as a survey of the number of families in the area covered by the Health Center’s School of Medicine Foundation ABC. However, as the sample was made to identify persons any procedure performed at home, involving the use of potentially contaminated materials, such as materials for fabrication of bandages, syringes and needles for insulin, among others. Such identification was performed by teams consisting of community workers and students of the Undergraduate Nursing FMABC.

The study was performed according to the Resolution No. 196/96 of the Ministry of Health, the study was submitted and approved by the Ethics Committee on Human Research FMABC (No. protocol 234/2008) affiliated with the National Research Council with Humans - CONEP.

To collect data, we used a questionnaire (Additional file 1) composed of 11 questions structured and semi-structured accessible to the general understanding of the studied population. The semi-structured questions were designed to allow greater spontaneity in the responses of the informant and meet the objectives proposed in this research. After the development of this instrument it was conducted a pilot test with people belonging to the people who were attending the Basic Network of Health Care and that would not be part of the sample.

The questionnaire was administered by the community employers and nursing students during the consultation with nursing supervision through interview question/answer. The exclusion criteria were patients who were not in the area served by the Basic Health Unit which covers the area of Pq Capuava. To be part of the research the patient had to be user of the primary service (SUS). The questionnaire was applied to the member or guardian of each family searched for collecting general data relating to the generation, handling, packaging and disposal of wastes consisting of biological materials generated at home searched. Issues of numbers 1 and 3 grouped several items related to public collection of household waste. The questions of 4, 5, 6, 7 and 8 were prepared to investigate the type of material (material bandage, gauze, cotton, needles, insulin or other materials drill-cutting, etc.) used to continue the treatment initiated in the hospital or in the Basic Health Units as well as the packaging and final destination to exempt these materials.

The question 9 was designed to verify the informant’s opinion if materials such as needles, syringes, sharps-drill materials, among others used in making applications and injections may cause the risk of disease transmission during contact with such materials.

Issues of numbers 10 and 11 are on the guidelines that the informant received or not health team about the handling and disposal of materials used in sick people.

The results are presented in tables in absolute and relative frequencies, or in text form to the small results. The processing and data analysis were performed using the statistical software Epi Info version 6.0. Data are presented in descriptive analysis, followed by hypothesis testing using parametric and univariate analyzes. We used the chi-square test, considering a significance level of 5% (p <0.05).

## Results

The age of 99 persons undergoing ranged from 20 to 60 years old, 68 were females and 31 were males, of whom 68 persons despised their waste by garbage collection, 28 walked to the basic units of health and three are ignored.

Regarding disposables, there were two categories, those that are needlestick scalpels, razor blades and insulin needle perforating and non-cutting which are the gauze, tape, feeding tube, cotton, among others. Only 32 people received counseling and 67 people received no guidance regarding the proper packaging and disposal of potentially infectious waste generated in their homes.

Table 1 shows persons that reported making use of disposable material, and most of them, people who uses the public collection to the final destination of these materials and people that refered disposables for the Basic Health Units. It was also observed subjects that reported not using disposable materials, most of them used the public collection, while a few walked to UBS, where your final destination is appropriate.

**Table 1:** Distribution of disposables, no-drill cutting and drill-cutting contaminated, according to the final destination: basic health unit and truck prefecture

**Variable**	**Use of the wasted material**		No-cutting material*		Cutting material*	
	**Yes**	**No**	**Yes**	**No**	**Yes**	**No**
	**p**	**χ**^**2**^	**p**	**χ**^**2**^	**p**	**χ**^**2**^
	**N(%)**	**N(%)**	**N(%)**	**N(%)**	**N(%)**	**N(%)**
Health basic unit	26(41.3)	2(6.9)	11(78.6)	17(21.8)	26(66.7)	2(3.5)
	0.002	9.5	<0.001	15.4	<0.001	41.7
Truck city hall	37(58.7)	27(93.1)	3(21.4)	61(78.2)	13(33.3)	55(96.5)
Total	63(100.0)	29(100.0)	14(100.0)	78(100.0)	39(100.0)	57(100.0)

Regarding cutting materials, less than a half of the subjects reported making use of such materials, the majority of them headed for the UBS material, while a small number of subjects used to collect public.

We note in Table 2 that great part of the population studied reported knowledge about the risk of disease transmission by potentially infectious materials generated at home environment, and almost the half use the public collection to the final destination of the waste. We also observed persons that send contaminated materials to the Basic Health Units, where storage and disposal are adequate. On the other hand, some subjects were unaware of the potential risks of such transmission, all of them use the public collection, for contempt of such waste.

**Table 2:** Knowledge of the population regarding the risk of disease transmission, possible contamination and receiving guidance from the health team

**Variable**	**Understand the risk of disease transmission**		**Worker more susceptible to contamination**		**Received guidance**	
	**Yes**	**No**	**Yes**	**No**	**Yes**	**No**
	**p**	**χ**^**2**^	**p**	**χ**^**2**^	**p**	**χ**^**2**^
	**N(%)**	**N(%)**	**N(%)**	**N(%)**	**N(%)**	**N(%)**
Health basic unit	28(38.4)	0(0.0)	28(36.8)	0(0.0)	25(78.1)	3(4.7)
	<0.01	10.6	0.02	5.3	<0.001	52.7
Truck city hall	45(61.6)	23(100.0)	48(63.2)	13(100.0)	7(21.9)	61(95.3)
Total	73(100.0)	23(100.0)	76(100.0)	13(100.0)	32(100.0)	64(100.0)

We also observed people that did not respond to the survey, however, great part of them reported knowing the potential risk of an individual being infected when handling potentially infectious materials generated at the household. Some subjects refered such material to Basic Health Units, while others use the public collection. However, a small part of the subjects reported that they had no knowledge of such contamination, also use the public collection.

Regarding the guidance received by the health team about the storage and disposal of waste generated at home, more than 30 people reported having received guidance on such procedures, the majority of them forwards materials potentially infectious generated at home, for Basic Health Units, while seven persons use the public collection. However, others reported not having had such guidance, the majority of them use the public collection.

Table 3 shows that some subjects reported using disposables, most of them reported not having received guidance on the proper handling of such materials, and a small part of the subjects did not respond. Regarding the use of non-cutting drill, some individuals reported use of such materials, and the majority referred to having received information on the proper handling of such waste.

**Table 3:** Distribution of materials about the neighborhood, user sharp objects and not drill-cutting

**Variable**	**Neighborhood**		**Uses disposable**		**Not drill-cutting material**	
	**Capuava and others neighborhodd**		**Yes**	**No**	**Yes**	**No**
	**p**	**χ**^**2**^	**p**	**χ**^**2**^	**p**	**χ**^**2**^
	**N(%)**	**N(%)**	**N(%)**	**N(%)**	**N(%)**	**N(%)**
Receive guidance	22(28.6)	10(45.5)	27(41.5)	5(16.7)	28(78.1)	4(6.8)
	0.2	1.52	0.03	4.6	<0.001	42.2
Did not received guidance	55(71.4)	12(54.5)	38(58.5)	25(83.3)	11(28.2)	55(93.2)
Total	77(100.0)	22(100.0)	65(100.0)	30(100.0)	39(100.0)	59(100.0)

According to Table 4 it is possible to identify that some subjects use drill cutting material and received guidance and another part did not use this material and received guidance, while some pople make use of drill cutting material and received no guidance as to its final destination and a great part of the population do not use and also received guidance. Regarding the risk of transmission, it was reported that 30 people know that there is a risk of disease transmission and receiving orientation, with two people did not know the risk of transmission. However, they were instructed concerning the final destination of contaminated materials, while more than a half are aware of the risk of disease transmission but did not receive counseling and almost all subjects did not know the risks and did not receive counseling. We also observed that some people know that other people who handle the waste as “garbage” may become contaminated and people received guidance and a small number of subjects did not know this way of contamination, but receive guidance, while another part of the subjcts know it can not infect others and receive guidance, already a great part of the subjects did not know and had not received guidance on storage and disposal of these contaminated materials.

**Table 4:** Distribution of material and drill-sharp knowledge of the population about the risk of disease transmission and possible contamination

**Variable**	**Cutting material**		**Disease transmission risk**		**Worker susceptible to infectious**	
	**Yes**	**No**	**Yes**	**No**	**Yes**	**No**
	**p**	**χ**^**2**^	**p**	**χ**^**2**^	**p**	**χ**^**2**^
	**N(%)**	**N(%)**	**N(%)**	**N(%)**	**N(%)**	**N(%)**
Received guidance	9(64.3)	22(27.8)	30(40.0)	2(8.7)	29(37.2)	2(15.4)
	0.007	7.11	0.01	6.48	0.22	1.49
Did not received guidance	5(35.7)	57(72.2)	45(60.0)	21(91.3)	49(62.8)	11(84.6)
Total	14(100.0)	79(100.0)	75(100.0)	23(100.0)	78(100.0	13(100.0)

We observe in Table 5 that some people refer disposables potentially infective route (perforating and non-cutting drill) generated at home for Basic Health Unit, and most of them uses 80.8% insulin needle, whereas a small part of the population make use of other potentially infectious materials, however one third of the subjects refer potentially infectious materials generated at home for collection public, whereas most 78, 8% use other types of materials, while some individuals using insulin needle. We observed that 40 individuals did not respond to search.

**Table 5:** Distribution concerning the final destination of the materials and guidance received from the health team

**Variable**	**Destination of materials**		**Received guidance**	
	**Health basic unit**	**City hall truck**	**Yes**	**No**
	**p**	**χ**^**2**^	**p**	**χ**^**2**^
	**N(%)**	**N(%)**	**N(%)**	**N(%)**
Others materials	5(19.2)	26(78.8)	7(25.9)	24(70.6)
	<0.001	18.3	<0.001	10.29
Insulin needle	21(80.8)	7(21.2)	20(74.1)	10(29.4)
Total	26(100.0)	33(100.0)	27(100.0)	34(100.0)

Regarding the guidance received by the health team, 27 people (27.2%) reported having received guidance on storage and disposal of this waste, most of these 74.1% use insulin needle, while 7 persons (25, 9%) using other types of materials. However, persons 34 (34.3%) reported not to have received such directions, that the majority (70.6%) uses other types of materials potentially infected, while 10 persons (29.4%) use insulin needle.

## Discussion

The profile of solid waste involves the quality of human and environmental health, because it contains a portion of each material from companies and establishments primarily residences and a broad spectrum of pathogens, as well as numerous toxic elements [5]. We also reported that the generating sources come from needlestick and dressings product.

Biological agents in the solid waste may be responsible for direct and indirect transmission of diseases, particularly those directly involved in handling this, as employees of the public collection, the garbage pickers who rummage looking for materials that may be marketed. These people are exposed to all types of contamination in these wastes materials [6]. Due to high unsanitary and dangerous this activity, aggravated possibly by living conditions that it presents [7]. Prominent among the risks identified in the process of garbage collection: mechanical (cuts, injuries, pedestrian accidents, serious falls), ergonomic (overexertion), biological (contact with pathogenic biological agents), chemical (toxic chemicals) and social (lack of training for service) [8].

Among the waste residues with microorganisms, it deserves special infectious waste and drill-cutting because they are the main causes of disease transmission to professionals who handle domestic garbage. The main cause of these accidents is the lack of information and awareness of the general population as can be seen in Tables 3 and 4, the lack of concern in separating contaminated materials, especially the drill cutting waste submitted to the home collection. As the RDC 306 materials used in services related to health care or feed, require special steps, such as management, segregation, packaging, identification and disposal of these materials [9].

The vast majority of the population still does not know how some materials can be harmful to human and environmental health [10-14]. Therefore, health services should emphasize programs in health care, issues involving environmental sanitation in order to raise awareness of the importance of proper storage of potentially infectious waste generated in your home to prevent possible risk of contamination through improper contact.

The processing of waste generated in the home environment, from its generation to its final destination, must respect the same rules of management described in the DRC 306, so they are not neglected and treated as harmful to the environment.

Through this study we suggest to the School Health Center, where the research was conducted, implementation of workshops for community health workers and the general population in order to enhance their knowledge about the storage and disposal of potentially infectious waste generated at home, thereby reducing the potential risk of disease transmission by improper management of these.

Thus, it is important to note that the occurrence of diseases transmitted through improper handling of potentially infectious waste generated at home can be avoided from the knowledge and awareness of the general public about the importance and responsibility that each citizen has in preserving human health and environmental. In this context the role of the nurse is critical in raising awareness and building knowledge of the population on these issues, since beyond its legal competence and training given to him for direct assistance to individuals, families and communities, adds to the their involvement in the daily context of the problems arising from the daily life of every citizen.

## Conclusion

The user populations of potentially infectious disposables generated at home know the proper ways of storage, final destination and the potential risk of an individual being infected when handling these materials. Therefore, it can be observed that people who use these materials not received guidance from the health team on the proper management of the same.

## Competing interests

The authors declare that they have no competing interests.

## Authors’ contributions

LCC, LMD, RF, LCA, VEV, LAA, VBCJ, DFS, FCG and FLAF participated in the acquisition of data and revision of the manuscript. LCC, LMD, RF, LCA, VEV, LAA, VBCJ, DFS and FLAF determined the design, interpreted the data and drafted the manuscript. All authors read and gave final approval for the version submitted for publication.

## Supplementary Material

Additional file 1 Questionário. Click here for file
