# Supplementary material for: Potentially hazardous waste produced at home
Source: Int Arch Med. 2013 Jun 27;6:27. doi: 10.1186/1755-7682-6-27 (PMC3718708; doi:10.1186/1755-7682-6-27)
Supplement: Additional file 1 — Questionário. [file 1755-7682-6-27-S1.docx]

QUESTIONÁRIO

Instrumento de coleta de dados que será aplicado ao responsável ou membro de famílias que utilizam materiais necessários para dar continuidade ao seu tratamento iniciado no hospital ou em Unidades Básicas de Saúde.

Prezado Sr. ou Sra. esta pesquisa pretende obter informações sobre a forma como são guardados e desprezados os materiais utilizados em sua casa, tais como: materiais de curativo, agulhas de insulina, agulhas de injeção ou outros materiais utilizados desta natureza. Desta forma esperamos contar com a sua colaboração respondendo as questões abaixo relacionadas. Asseguramos que sua identificação bem como de sua família será mantida em sigilo. Desde já agradecemos a sua colaboração.

Identificação - Data / /

Município: Bairro:

Unidade Básica de Atenção à Saude:

1- Tipo de construção da moradia:

( ) madeirite ( ) madeira ( ) alvenaria ( ) apartamento

( ) outro tipo____________________

2- Número de cômodos:

( ) 01 cômodo ( ) 02 cômodos ( ) 03 cômodos ( ) 04 cômodos

( ) mais de 04

3- Número de pessoas que trabalham:

( ) 01 pessoa ( ) 02 pessoas ( ) 03 pessoas ( ) todos da família

outros________________________________________________________

4- Tipo de trabalho:

( ) com registro na carteira profissional ( ) trabalho informal

5- Remuneração mensal da família:

( ) menos que um salário mínimo ( ) um salário mínimo

( ) dois salários mínimos ( ) três salários mínimos

( ) quatro salários mínimos ( ) cinco salários mínimos

( ) de seis a dez salários mínimos ( ) de onze a quinze salários mínimos

( ) de dezesseis a vinte salários mínimos ( ) mais de vinte salários

(Salário mínimo vigente = R$ ------)

6- Este local no qual o Sr. (Sra.) mora tem rede de abastecimento de água?

( ) SIM ( ) NÃO

7- Se a resposta anterior for negativa (não), qual é a procedência da água que a família utiliza?

( ) do rio ( ) do vizinho ( ) caminhão da prefeitura outro lugar_________

8- Este local no qual o Sr. (Sra.) mora tem rede de esgoto?

( ) SIM ( ) NÃO

9- Se a resposta for negativa (não), em que local são desprezados a água utilizada para limpeza (lavagem de roupas, louças de cozinha...) e dejetos humanos (fezes, urina ou outras secreções eliminadas pelo corpo)?

( ) fossa ( ) a céu aberto Outro lugar___________________________

10- Existe coleta pública do lixo produzido em sua casa?

( ) SIM ( ) NÃO

11- Se a resposta for positiva (sim), quantas vezes é realizada esta coleta pública do lixo?

( ) a cada quinze dias ( ) uma vez/semana ( ) duas vezes/semana

( ) 3 vezes/semana outros ______________________________________

12- Se a resposta n 7 for negativa (não), em que local o Sr. ou Sra. despreza o lixo produzido em sua casa?

( ) enterro no quintal ( ) terreno baldio ( ) no rio ( ) no lixão

Outro lugar_________________________________________________

13- Existe alguém com alguma doença na família?

( ) SIM ( ) NÃO

14- Se a resposta anterior for positiva (sim), quantas pessoas estão doentes?

( ) uma pessoa ( ) duas pessoas ( ) três pessoas ( ) mais de três

15- Esta ou estas pessoas utilizam algum material descartável (material de curativo, agulhas de insulina, outros tipos de agulha ou outro tipo de material) proveniente de algum serviço de saúde para o tratamento da doença?

( ) SIM ( ) NÃO

16- Qual ou quais são os materiais utilizados?

( ) agulha de insulina ( ) gazes ( ) bisturi ( ) esparadrapo

( ) agulha de injeção ( ) sonda para alimentação

( ) sonda para eliminação de urina Outros__________________________

17- De que forma são desprezados os materiais não perfurocortantes (gazes, algodão, esparadrapo...)?

( ) saco de lixo ( ) saco de lixo branco leitoso ( ) não são embalados

( ) lata de leite em pó vazia

( ) caixa de papelão ( ) no saco de lixo Outro recipiente_______________

18- De que forma são desprezados os materiais perfurocortantes (agulhas, bisturis, gilete ...)?

( ) no saco de lixo comum ( ) saco de lixo branco leitoso

( ) caixa de papelão ( ) lata de leite em pó vazia ( ) Outro______________

19- Qual é o destino final desses materiais utilizados em sua casa?

( ) terreno baldio ( ) lixão ( ) levado pelo caminhão da prefeitura

( ) o pessoal da unidade de saúde busca Outro______________________

20- Na sua opinião estes materiais após serem utilizados (agulhas de insulina, agulha de injeção, gazes, esparadrapo...), possuem algum risco de transmissão de doença?

( ) não sei ( ) sim ( ) não

Se a resposta for negativa (não) ou afirmativa (sim), por que?

_____________________________________________________________

21- O Sr. ou Sra. recebe ou recebeu alguma orientação sobre o manuseio destes materiais pela equipe de saúde?

( ) SIM ( ) NÃO

22- Se a resposta anterior for positiva (sim), especifique qual foi o tipo de orientação?

( ) não desprezar agulhas no saco de plástico e sim em recipiente resistente

( ) evitar de entrar em contato com o material utilizado

( ) não desprezar junto com o lixo comum

Outras orientações____________________________________________
